# Cardio–Renal Diseases Are Independent Risk Factors of Severe Human Metapneumovirus Infection Among Patients Without Chronic Airway Diseases

**DOI:** 10.1002/jmv.70812

**Published:** 2026-01-29

**Authors:** Wang Chun Kwok, Isaac Sze Him Leung, Chun Ka Emmanuel Wong, James Chung Man Ho, David Chi Leung Lam, Mary Sau Man Ip, Shuk Man Ngai, Kelvin Kai Wang To, Desmond Yat Hin Yap

**Affiliations:** ^1^ Division of Respiratory Medicine, Department of Medicine, School of Clinical Medicine, LKS Faculty of Medicine, Queen Mary Hospital The University of Hong Kong Hong Kong; ^2^ Department of Statistics The Chinese University of Hong Kong Shatin New Territories Hong Kong; ^3^ Division of Cardiology, Department of Medicine, School of Clinical Medicine, LKS Faculty of Medicine, Queen Mary Hospital The University of Hong Kong Hong Kong; ^4^ Department of Microbiology, School of Clinical Medicine, LKS Faculty of Medicine The University of Hong Kong Hong Kong; ^5^ Division of Nephrology, Department of Medicine, School of Clinical Medicine, LKS Faculty of Medicine, Queen Mary Hospital The University of Hong Kong Hong Kong

**Keywords:** human metapneumovirus, mortality, respiratory failure, risk factor, secondary bacterial pneumonia

## Abstract

Human metapneumovirus (hMPV) causes mild and self‐limiting disease in adults. However, the risk factors for serious adverse outcomes following hMPV infection in adult patients without preexisting chronic airway diseases remain poorly understood. We conducted a territory‐wide retrospective study on adult patients (aged ≥ 18 years) without chronic airway diseases hospitalized for hMPV infections between January 1, 2016 and June 30, 2023 in Hong Kong. We assessed the incidence and risk factors for in‐patient mortality, severe respiratory failure (SRF), secondary bacterial pneumonia and acute kidney injury (AKI) were assessed. A total of 1552 eligible adult patients without chronic airway diseases hospitalized for hMPV infections were analyzed. Within the index admission, 92 (5.9%) patients died. Ischemic heart disease (IHD) was associated with increased risks of SRF [adjusted odds ratio (aOR) 2.00 (95% CI 1.48–2.71), *p* < 0.001]. IHD, heart failure (HF), and history of ischemic stroke were significant predictors for AKI [aOR 1.51 (95% CI 1.12–2.04), 2.87 (95% CI 2.14–3.85), and 1.47 (95% CI = 1.12–1.93), *p* = 0.007, < 0.001, and 0.005, respectively). Patients with end‐stage kidney disease (ESKD) requiring renal replacement therapy (RRT) were at increased risk of in‐patient mortality [aOR 6.36 (95% CI 2.34–17.26), *p* < 0.001] and SRF [aOR 8.80 (95% CI 3.84–20.16), *p* < 0.001]. The presence of cardiovascular diseases and ESKD requiring RRT is a strong predictor of severe in‐hospital outcomes among adult patients without chronic airway diseases who are hospitalized for hMPV infections.

## 1 Introduction

Human metapneumovirus (hМРV) was first discovered in 2001 as a viral cause of respiratory tract infections [1]. hΜРV is a member of the family Pneumoviridae, which comprises large enveloped negative‐sense RNA viruses [2]. hMPV is an enveloped virus with a nonsegmented negative‐sense RNA genome. The surface glycoproteins of hMPV are crucial for infection. The fusion (F) protein mediates viral entry by promoting membrane fusion, while the G glycoprotein is responsible for initial attachment to host cell receptors. The F protein facilitates the merging of viral and cellular membranes, enabling genome entry, while the G protein helps the virus bind to host cells, aiding infection initiation. Together, these glycoproteins are vital for viral attachment, entry, and subsequent replication within host tissues [3].

hΜPV can cause upper and lower respiratory tract infection in patients of all age groups, but symptomatic disease mostly occurs in young children or older adults [4]. In adult patients, hMPV was detected in about 4% of hospitalized adults with community‐acquired pneumоnia [5]. The clinical manifestations of hMPV in adults include cough, nasal congestion, rhinorrhoea, dyspnea, hoarseness, and wheezing [6]. hMPV has been demonstrated to cause lower respiratory tract infections in hospitalized adult patients, especially elderly people with preexisting medical comorbidities, such as chronic respiratory diseases and hypertension [7]. In one study, 30% of the patients had bacterial coinfection while the need for mechanical ventilation and/or the hospital death was observed in almost 20% of the patients [7]. Another French study also suggested that the elderly and patients with chronic conditions were mostly affected by hMPV infections and were responsible for frequent cardiac and pulmonary complications [8]. Of note, hMPV was reported to cause exacerbations of chronic obstructive pulmonary disease (COPD) [9, 10, 11] and asthma [12, 13, 14].

Currently, the treatment of hMPV is mainly supportive with very limited evidence for ribavirin treatment based on in vitro studies [15]. In recent years, breakthroughs in identifying the structure of the viral fusion (F) protein may bring about advancements in vaccine development and therapeutics, which could serve as a target for future vaccines and drugs [16, 17, 18, 19, 20, 21, 22, 23]. While hMPV infections in adults are mostly mild and self‐limiting [24], it is crucial to identify the target patient groups for consideration of future vaccination and treatment once they become available, as massive vaccination and liberal use of novel treatment such as neutralizing antibodies [21] will incur a significant burden on healthcare resources and costs.

## 2 Methods

This was a territory‐wide retrospective study to examine the risk factors for mortality and serious clinical outcomes in adult patients hospitalized for hMPV infections among patients who did not have underlying chronic airway diseases (asthma, COPD, asthma/COPD overlap (ACO) and bronchiectasis). Patients with chronic airway diseases were excluded, as the primary outcomes of the study could be related to hMPV infections as well as the underlying chronic airway diseases. Adult patients admitted to public hospitals in Hong Kong for hMPV infection between January 1, 2016 and June 30, 2023 were included. Patients with coexisting respiratory tract viral infections were excluded. Patients were identified from the Clinical Data Analysis and Reporting System (CDARS) of Hospital Authority (HA) by the principal diagnosis with the International Classification of Diseases, Ninth Revision (ICD‐9) codes 0789.89 or 466.19. CDARS is an electronic health record database managed by the HA, a public healthcare service provider that covered > 90% of the Hong Kong population since 1993 [25, 26, 27]. The study was approved by the Institutional Review Board (IRB) of the University of Hong Kong and the HA Hong Kong West Cluster (UW 24‐137). Patient informed consent was waived for this retrospective study by the IRB, as it involved no active patient recruitment and all the data were de‐identified. The study was conducted in compliance with the Declaration of Helsinki.

### 2.1 Outcome Measurements and Statistical Analysis

The co‐primary outcomes included: (1) death during hospitalization, (2) severe respiratory failure (SRF) requiring invasive or noninvasive mechanical ventilation, (3) secondary bacterial pneumonia, and (4) acute kidney injury (AKI). AKI was defined according to the RIFLE criteria [28]. Secondary bacterial pneumonia was identified by ICD‐9 diagnostic code of 481 or 482, or by the growth of pathogenic organisms in respiratory specimens during the same admission episode. The following covariates were assessed as potential risk factors associated with the outcomes: age and Charlson Comorbidity Index (CCI) as continuous variables; gender; history of malignancy; underlying diabetes mellitus (DM), ischemic heart disease (IHD), ischemic stroke, heart failure (HF), and underlying kidney diseases as categorical variables. For underlying kidney diseases, patients were further subclassified into those with end‐stage kidney disease (ESKD) requiring renal replacement therapy (RRT) and those with CKD [defined as estimated glomerular filtration rate (eGFR) < 60 mL/min/1.73 m^2^] [29]; baseline eGFR was also assessed as a continuous variable.

Descriptive tables and illustrative figures were created to present the incidence rates of severe in‐hospital outcomes. Demographic and clinical data were described as actual frequency or mean ± standard deviation (SD), or median [interquartile range] as appropriate. Continuous variables were compared by independent *t*‐test or Mann–Whitney *U* tests, as appropriate.

Risk factors for adverse clinical outcomes in patients hospitalized for hMPV infections were first assessed by univariate then multivariable analyses. Factors with a *p* < 0.1 in the univariate analysis were included in the multivariable model using backward selection.

Data analyses were performed using the 28th version of the SPSS statistical package. For all statistical analyses, statistical significance was assessed at an *α* level of 0.05. This report followed the STROBE and RECORD reporting guidelines.

## 3 Results

### 3.1 Patients' Characteristics

**Figure 1:**
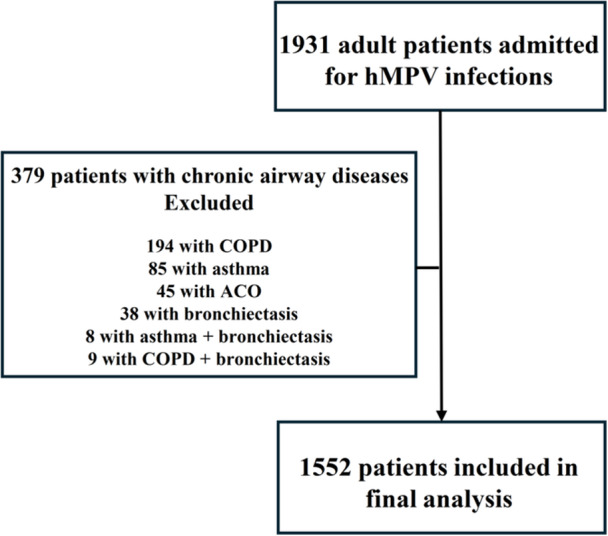
Patient selection flow chart.

A total of 1931 adult patients were hospitalized for hMPV infections in public hospitals in Hong Kong between January 1, 2016 and June 30, 2023. Of these, 379 patients with asthma, COPD, ACO or bronchiectasis were excluded (Figure 1). Consequently, 1552 patients were included in the final analysis. Among the 1552 included patients, 884 (57.0%) were female, with a mean age of 73.4 ± 18.2 years. The mean CCI was 5.08 ± 2.71. Patients' characteristics are summarized in Table 1.

**Table 1:** Baseline clinical characteristics of the patients included.

Number of subjects *N*	1552	
Age, years (mean (SD))	73.4	(18.2)
Female (*N*, (%))	884	(57.0)
History of malignancies (*N*, (%))	219	(14.1)
Diabetes mellitus (*N*, (%))	432	(27.8)
Ischemic heart disease (*N*, (%))	244	(15.7)
Heart failure (*N*, (%))	282	(18.2)
History of stroke (*N*, (%))	305	(19.7)
Peripheral vascular disease (*N*, (%))	23	(1.5)
Dementia (*N*, (%))	334	(21.5)
Connective tissue disease (*N*, (%))	218	(14.0)
CKD (*N*, (%))	671	(43.2)
ESKD requiring RRT (*N*, (%))	30	(1.9)
CCI (mean (SD))	5.08	(2.71)
eGFR, mL/min/1.73 m^2^ (mean (SD))	65.3	(28.1)
Influenza vaccine (*N*, (%))	500	(32.2)
Pneumococcal conjugated vaccines (*N*, (%))	200	(12.9)
Pneumococcal polysaccharide vaccine (*N*, (%))	59	(3.8)
Ethnicity (*N*, (%))		
Chinese	1516	(97.7)
Southeast Asian	10	(0.6)
South Asian	4	(0.3)
Caucasian	3	(0.2)
Others	19	(1.2)

Abbreviations: CKD, chronic kidney disease; CCI, Charlson Comorbidity Index; ESKD, end‐stage kidney disease; RRT, renal replacement therapy.

### 3.2 Risk Factors for Severe In‐Hospital Outcomes

**Figure 2:**
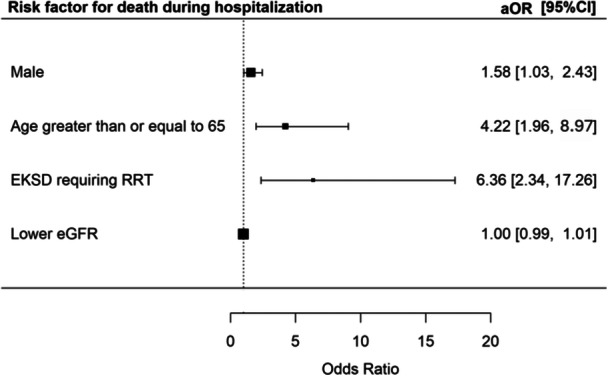
Risk factors for death during hospitalization.

A total of 92 (5.9%) patients died during the index admission. Risks of mortality during hospitalization were increased in male patients [aOR 1.58 (95% CI 1.03–2.43), *p* = 0.037], patients age ≥ 65 [aOR 4.22 (95% CI 1.96–8.97), *p* < 0.001], and EKSD patients requiring RRT [aOR 6.36 (95% CI 2.34–17.26), *p* < 0.001] (Figure 2).

**Figure 3:**
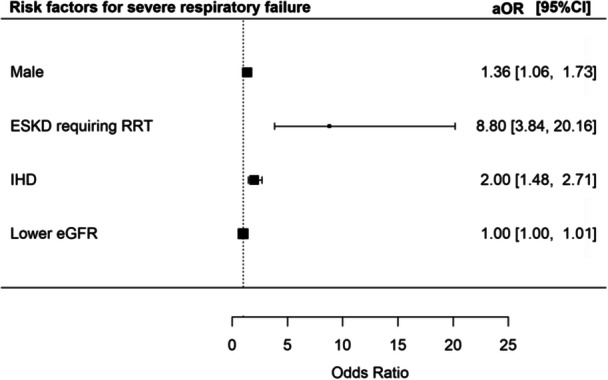
Risk factors for severe respiratory failure.

A total of 349 (22.5%) patients developed SRF. The risk of SRF was increased in male patients [aOR 1.36 (95% CI 1.06–1.73), *p* = 0.016], patients with IHD [aOR 2.00 (95% CI 1.48–2.71), *p* < 0.001], and patients with EKSD requiring RRT [aOR 8.80 (95% CI 3.84–20.16), *p* < 0.001] (Figure 3).

In total, 1058 (68.2%) of the patients developed secondary bacterial pneumonia, with an increased risk in patients aged ≥ 65 [aOR 1.41 (95% CI 1.11–1.80), *p* = 0.005].

**Figure 4:**
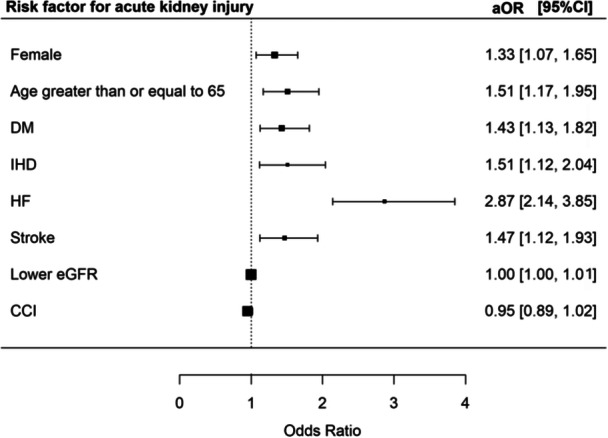
Risk factor for acute kidney injury.

In total, 660 (42.5%) of the patients developed AKI. The risk of AKI was increased in female patients [aOR 1.33 (95% CI 1.07–1.65), *p* = 0.010], patients age ≥ 65 [aOR 1.51 (95% CI 1.17–1.95), *p* < 0.001], patients with DM [aOR 1.43 (95% CI 1.13–1.82), *p* = 0.003], patients with IHD [aOR 1.51 (95% CI 1.12–2.04), *p* = 0.007], patients with HF [aOR 2.87 (95% CI 2.14–3.85), *p* < 0.001], and patients with a history of stroke [aOR 1.47 (95% CI 1.12–1.93), *p* = 0.005] (Figure 4).

### 3.3 Subgroup Analysis

As older age is a risk factor for severe hMPV infections, a subgroup analysis was performed for patients aged < 65 (*n* = 410) and ≥ 65 (*n* = 1142).

Among patients aged < 65 years of age, 9 (2.2%) patients died in the index admission, 111 (27.1%) developed SRF, 253 (61.7%) developed secondary bacterial pneumonia, and 121 (29.5%) developed AKI.

Among patients aged ≥ 65, 83 (7.3%) patients died in the index admission, 238 (20.8%) developed SRF, 805 (70.5%) had secondary bacterial pneumonia, and 539 (47.2%) developed AKI. The risk factors for the severe in‐hospital outcomes among patients aged < 65 and ≥ 65 years were summarized in Table S1.

## 4 Discussion

In this study, we demonstrated that medical comorbidities, especially cardiovascular and renal diseases, are risk factors for adults without chornic airway diseases hospitalized for severe hMPV infections. These findings provide insight for potential patient selection for future hMPV vaccination and treatment when these agents soon become available.

In this study, we demonstrated that adverse in‐hospital outcomes were common among adults hospitalized for hMPV infections. While hMPV infection in adults is often considered mild and self‐limiting, this may not apply to older adults with more medical comorbidities who require hospitalization, even in the absence of chronic airway diseases. Various cardiovascular and renal diseases were associated with different adverse outcomes, which aligns with literature suggesting chronic medical conditions as risk factors for severe hMPV infection [7]. Our study findings presented here illustrated this phenomenon better by segregating these chronic medical diseases into specific diseases, which allowed a better understanding on the risk stratification. In this context, ESKD requiring RRT emerged as a strong risk factor for in‐patient mortality in adults hospitalized for hMPV infections. Patients with ESKD requiring RRT were demonstrated to have an increased mortality during index admission and SRF. It is well recognized that patients with ESKD are immunocompromised, rendering them highly susceptible to serious infections and complications [30]. A similar phenomenon was observed in the coronavirus disease in 2019 (COVID‐19) [31]. A Japanese study suggested that the risk of death and renal function decline was increased among ESKD patients requiring RRT who had COVID‐19, but not in nondialysis‐dependent CKD patients. The risk of severe outcomes from viral infections such as hMPV in RRT‐dependent patients should not be overlooked.

hMPV is a respiratory virus that was identified relatively recently, though scientists believe that it has been causing respiratory tract infections for decades [32, 33]. Since its discovery in 2001, substantial progress has been made in vaccination and therapeutic research. However, there is a need for case identification in order to design appropriate vaccination and treatment strategies, given the fact that novel vaccines and therapeutic agents can be costly. Our findings could help identify risk groups that are more likely to benefit from receiving vaccination and viral‐specific treatment.

Underlying cardiovascular diseases are common across ethnic groups and have been suggested to be associated with increased risks of complications in patients having various viral respiratory tract infections [34, 35, 36]. This phenomenon is also observed among adult patients hospitalized for hMPV infections. Another high‐risk subgroup that was identified to be associated with severe hMPV infections is those who had ESKD requiring RRT. Studies on other viral infections, such as COVID‐19, suggest that these patients have prolonged viral shedding [37] and more severe disease [38]. Immune dysfunction also predisposes patients with CKD to develop severe infections [39]. However, the number of EKSD patients requiring RRT in our cohort is relatively small. Despite a significant result, this finding should be interpreted with caution, and a validation in a larger cohort is warranted.

The high rate of severe in‐hospital outcomes concurs with previous reports that elderly patients and those with medical comorbidities are at risk of severe disease, including bacterial coinfection and respiratory failure. hMPV should not be considered as a mild, self‐limiting infection among hospitalized patients. These patients are at risk of developing various severe in‐hospital outcomes, including mortality. Close monitoring of respiratory status and early initiation of antibiotics are warranted, as secondary bacterial infection is common. Apart from respiratory complications, we also overserved a high incidence of AKI, similar to that seen in COVID‐19 and influenza infections [40]. This highlights the importance of monitoring other organ functions in patients hospitalized for hMPV, especially elderly patients with medical comorbidities, who may develop AKI due to sepsis, dehydration from anorexia, and also drug‐induced AKI.

Our study has several limitations. First, it was conducted in Hong Kong, the majority of the patients are Chinese, and the generalizability to other populations remains to be determined. Some demographic data, such as ethnicity, were missing and were handled by multiple imputation. We also performed subgroup analyses to ensure our data is robust across different age groups. Second, we did not analyze the granular details of secondary bacterial pneumonia. Third, only hospitalized adults were included; non‐hospitalized subjects were not analyzed, which may limit the assessment of hMPV infection as a whole and likely elevates the observed complication rate. Nevertheless, it is important to analyze those hospitalized subjects incur much more healthcare burden than nonhospitalized subjects, warranting dedicated assessment. While these inherent limitations may affect our results, it is important to appreciate that our data are derived from a territory‐wide electronic health record system that captures comprehensive clinical information for all adults hospitalized for adenoviruses or seasonal influenza infection during the study period, providing a good representation of real‐world data.

In summary, our study presented the incidence and risk factors for severe hMPV infections in adult patients hospitalized without chronic airway diseases. The presence of cardiovascular and renal comorbidities, especially among elderly patients, should warrant timely monitoring and management of severe in‐hospital outcomes.

## 5 Conclusions

The presence of cardiovascular diseases and ESKD requiring RRT was associated with an increased risk of severe in‐hospital outcomes among adult patients without chronic airway diseases who are hospitalized for hMPV infections.

## Author Contributions

Conceptualization: Wang Chun Kwok, Desmond Yat Hin Yap, Isaac Sze Him Leung. Data curation: Wang Chun Kwok, Isaac Sze Him Leung, Shuk Man Ngai. Formal analysis: Wang Chun Kwok, Isaac Sze Him Leung, Shuk Man Ngai. Investigation: Wang Chun Kwok, Isaac Sze Him Leung, Shuk Man Ngai, Desmond Yat Hin Yap, Chun Ka Emmanuel Wong. Methodology: Wang Chun Kwok, Isaac Sze Him Leung, Shuk Man Ngai, Chun Ka Emmanuel Wong. Project administration: Wang Chun Kwok, Isaac Sze Him Leung, Shuk Man Ngai, Chun Ka Emmanuel Wong. Resources: Wang Chun Kwok. Software: Wang Chun Kwok, Isaac Sze Him Leung, Shuk Man Ngai, Desmond Yat Hin Yap, Chun Ka Emmanuel Wong. Supervision: James Chung Man Ho, David Chi Leung Lam, Mary Sau Man Ip, Kelvin Kai Wang To, Desmond Yat Hin Yap. Validation: James Chung Man Ho, David Chi Leung Lam, Mary Sau Man Ip, Kelvin Kai Wang To, Desmond Yat Hin Yap. Visualization: Wang Chun Kwok, Desmond Yat Hin Yap. Writing – original draft preparation: Wang Chun Kwok, Desmond Yat Hin Yap. Writing – review and editing: James Chung Man Ho, David Chi Leung Lam, Mary Sau Man Ip, Kelvin Kai Wang To, Desmond Yat Hin Yap.

## Ethics Statement

The study was approved by the Institutional Review Board (IRB) of the University of Hong Kong and Hospital Authority Hong Kong West Cluster (UW 24‐137). The study was conducted in compliance with the Declaration of Helsinki.

## Consent

Patient informed consent was waived in this retrospective study by the IRB as it is a retrospective study without active patient recruitment while the data was already de‐identified.

## Conflicts of Interest

The authors declare no conflicts of interest.

## Supporting information


**Supplementary Table 1:** Risk factors for severe in‐hospital outcomes among HMPV patients in the whole cohort. **Supplementary Table 2:** Risk factors for severe in‐hospital outcomes among HMPV patients age < 65. **Supplementary Table 3:** Risk factors for severe in‐hospital outcomes among HMPV patients ≥ 65.

## Data Availability

All available data are presented in the manuscript and no additional data will be provided. The ethics committee of the institute did not permit the authors to share the data to third party due to data protection privacy.
