# Supplementary material for: Cardio–Renal Diseases Are Independent Risk Factors of Severe Human Metapneumovirus Infection Among Patients Without Chronic Airway Diseases
Source: J Med Virol. 2026 Jan 29;98(2):e70812. doi: 10.1002/jmv.70812 (PMC12853410; doi:10.1002/jmv.70812)
Supplement: Supplementary file 1 — Supplementary Table 1: Risk factors for severe in‐hospital outcomes among HMPV patients in the whole cohort. Supplementary Table 2: Risk factors for severe in‐hospital outcomes among HMPV patients age < 65. Supplementary Table 3: Risk factors for severe in‐hospital outcomes among HMPV patients ≥ 65. [file JMV-98-e70812-s001.docx]

**Supplementary Table 1 Risk factors for severe in-hospital outcomes among HMPV patients in the whole cohort**

|  | **Univariate analysis** | | | **Multi-variable analysis†** | | |
| --- | --- | --- | --- | --- | --- | --- |
| **Death during hospitalization** | | | | | | |
|  | **OR** | **95% CI** | **p-value** | **aOR** | **95% CI** | **p-value** |
| Male* | 1.62 | 1.06 – 2.48 | 0.025 | 1.58 | 1.03 – 2.43 | 0.037 |
| Age ≥ 65* | 3.49 | 1.74 - 701 | <0.001 | 4.22 | 1.96 – 8.97 | <0.001 |
| ESKD requiring RRT* | 4.17 | 1.66 – 10.48 | 0.004 | 6.36 | 2.34 – 17.26 | <0.001 |
| Lower eGFR | 1.01 | 1.00 -1.02 | 0.036 | 1.00 | 0.99 – 1.01 | 0.836 |
| **Severe respiratory failure** | | | | | | |
|  | **OR** | **95% CI** | **p-value** | **aOR** | **95% CI** | **p-value** |
| Male* | 1.43 | 1.12 – 1.81 | 0.004 | 1.36 | 1.06 – 1.73 | 0.016 |
| ESKD requiring RRT* | 10.05 | 4.43 – 22.78 | <0.001 | 8.80 | 3.84 – 20.16 | <0.001 |
| IHD* | 2.02 | 1.50 – 2.72 | <0.001 | 2.00 | 1.48 – 2.71 | <0.001 |
| Lower eGFR | 1.01 | 1.00 – 1.01 | 0.019 | 1.00 | 1.00 - 1.01 | 0.224 |
| **Secondary bacterial pneumonia** | | | | | | |
|  | **OR** | **95% CI** | **p-value** | **aOR** | **95% CI** | **p-value** |
| Age ≥ 65* | 1.48 | 1.17 – 1.88 | 0.001 | 1.41 | 1.11 – 1.80 | 0.005 |
| IHD | 1.31 | 0.97 – 1.78 | 0.081 | 1.10 | 0.79 – 1.53 | 0.567 |
| HF | 1.45 | 1.08 – 1.94 | 0.012 | 1.34 | 0.99 – 1.80 | 0.055 |
| Lower eGFR | 1.01 | 1.00 – 1.01 | 0.013 | 1.00 | 1.00 – 1.01 | 0.811 |
| CCI | 1.07 | 1.03 – 1.12 | <0.001 | 1.04 | 0.98 – 1.10 | 0.216 |
| **AKI** | | | | | | |
|  | **OR** | **95% CI** | **p-value** | **aOR** | **95% CI** | **p-value** |
| Female* | 1.41 | 1.15 – 1.74 | <0.001 | 1.33 | 1.07 – 1.65 | 0.010 |
| Age ≥ 65* | 2.14 | 1.68 – 2.72 | <0.001 | 1.51 | 1.17 – 1.95 | <0.001 |
| DM* | 1.83 | 1.46 – 2.29 | <0.001 | 1.43 | 1.13 – 1.82 | 0.003 |
| IHD* | 2.21 | 1.67 – 2.92 | <0.001 | 1.51 | 1.12 – 2.04 | 0.007 |
| HF* | 2.96 | 3.00 – 5.23 | <0.001 | 2.87 | 2.14 – 3.85 | <0.001 |
| History of ischaemic stroke* | 1.85 | 1.44 – 2.38 | <0.001 | 1.47 | 1.12 – 1.93 | 0.005 |
| Lower eGFR | 1.01 | 1.01 – 1.01 | <0.001 | 1.00 | 1.00 – 1.01 | 0.97 |
| CCI | 1.16 | 1.11 – 1.20 | <0.001 | 0.95 | 0.89 – 1.02 | 0.157 |

*: Factors that are statistically significant in multi-variable analysis

^†:^ Adjustment done for factors with p <0.10 in univariate analysis

DM = diabetes mellitus; IHD = ischaemic heart disease; eGFR = estimated glomerular filtration rate; RRT = Renal replacement therapy; CCI = Charlson comorbidity index; ESKD = End-stage kidney disease; AKI = Acute kidney injury

**Supplementary Table 2 Risk factors for severe in-hospital outcomes among HMPV patients age < 65**

|  | **Univariate analysis** | | | **Multivariate analysis**^†^ | | |
| --- | --- | --- | --- | --- | --- | --- |
| **Death during hospitalization** | | | | | | |
|  | **OR** | **95% CI** | **p-value** | **aOR** | **95% CI** | **p-value** |
| History of ischaemic stroke | 3.96 | 0.78 – 19.98 | 0.096 | 3.05 | 0.56 – 16.54 | 0.196 |
| ESKD requiring RRT* | 6.45 | 1.25 – 33.43 | 0.026 | 6.45 | 1.25 – 33.43 | 0.026 |
| **Severe respiratory failure** | | | | | | |
|  | **OR** | **95% CI** | **p-value** | **aOR** | **95% CI** | **p-value** |
| Male* | 1.98 | 1.27 – 3.07 | 0.003 | 2.18 | 1.38 – 3.43 | <0.001 |
| ESKD requiring RRT | 5.06 | 1.94 – 13.20 | <0.001 | 2.38 | 0.83 – 6.79 | 0.106 |
| DM | 1.79 | 1.02 – 3.13 | 0.043 | 1.12 | 0.59 – 2.12 | 0.730 |
| Lower eGFR * | 1.02 | 1.01 – 1.02 | <0.001 | 1.02 | 1.01 - 1.02 | <0.001 |
| **AKI** | | | | | | |
|  | **OR** | **95% CI** | **p-value** | **aOR** | **95% CI** | **p-value** |
| Older age | 1.03 | 1.01 – 1.05 | 0.002 | 1.02 | 1.00 – 1.04 | 0.085 |
| DM | 2.29 | 1.32 – 3.95 | 0.003 | 1.68 | 0.92 – 3.09 | 0.093 |
| HF | 2.53 | 1.07 – 6.00 | 0.036 | 1.39 | 0.54 – 3.59 | 0.50 |
| History of ischaemic stroke* | 4.42 | 2.02 – 9.67 | <0.001 | 3.91 | 1.74 – 8.80 | 0.001 |
| Lower eGFR* | 1.02 | 1.01 – 1.02 | <0.001 | 1.01 | 1.00 – 1.02 | 0.032 |

*: Factors that are statistically significant in multi-variable analysis

^†:^ Adjustment done for factors with p <0.10 in univariate analysis

DM = diabetes mellitus; eGFR = estimated glomerular filtration rate; RRT = Renal replacement therapy; ESKD = End-stage kidney disease; AKI = Acute kidney injury

**Supplementary Table 3 Risk factors for severe in-hospital outcomes among HMPV patients ≥ 65**

|  | **Univariate analysis** | | | **Multivariate analysis**^†^ | | |
| --- | --- | --- | --- | --- | --- | --- |
| **Death during hospitalization** | | | | | | |
|  | **OR** | **95% CI** | **p-value** | **aOR** | **95% CI** | **p-value** |
| ESKD requiring RRT* | 7.61 | 2.18 – 26.55 | 0.001 | 7.61 | 2.18 – 26.55 | 0.001 |
| **Severe respiratory failure** | | | | | | |
|  | **OR** | **95% CI** | **p-value** | **aOR** | **95% CI** | **p-value** |
| ESKD requiring RRT* | 39.61 | 5.04 – 310.98 | <0.001 | 37.77 | 4.75 – 300.21 | <0.001 |
| IHD* | 2.50 | 1.80 – 3.46 | <0.001 | 2.47 | 1.77 – 3.43 | <0.001 |
| Lower eGFR | 1.01 | 1.00 – 1.01 | 0.053 | 1.00 | 1.00 - 1.01 | 0.270 |
| **Secondary bacterial pneumonia** | | | | | | |
|  | **OR** | **95% CI** | **p-value** | **aOR** | **95% CI** | **p-value** |
| Older age* | 1.02 | 1.00 – 1.03 | 0.030 | 1.02 | 1.00 – 1.03 | 0.012 |
| Male* | 1.30 | 1.01 – 1.69 | 0.046 | 1.45 | 1.10 – 1.91 | 0.009 |
| HF* | 1.45 | 1.05 – 1.99 | 0.023 | 1.39 | 1.00 – 1.94 | 0.049 |
| History of ischaemic stroke* | 1.74 | 1.26 – 2.40 | <0.001 | 1.60 | 1.15 – 2.22 | 0.005 |
| CCI | 1.09 | 1.02 – 1.16 | 0.009 | 1.01 | 0.94 – 1.09 | 0.850 |
| **AKI** | | | | | | |
|  | **OR** | **95% CI** | **p-value** | **aOR** | **95% CI** | **p-value** |
| Female | 1.49 | 1.18 – 1.89 | <0.001 | 1.14 | 0.87 – 1.47 | 0.341 |
| Older age* | 1.04 | 1.03 – 1.06 | <0.001 | 1.04 | 1.03 – 1.06 | <0.001 |
| DM* | 1.55 | 1.21 – 1.99 | <0.001 | 1.34 | 1.03 – 1.75 | 0.030 |
| IHD* | 2.02 | 1.49 – 2.73 | <0.001 | 1.51 | 1.09 – 2.09 | 0.013 |
| HF* | 3.68 | 2.73 – 4.97 | <0.001 | 3.07 | 2.25 – 4.18 | <0.001 |
| History of ischaemic stroke | 1.43 | 1.09 – 1.88 | 0.010 | 1.23 | 0.92 – 1.64 | 0.162 |

*: Factors that are statistically significant in multi-variable analysis

^†:^ Adjustment done for factors with p <0.10 in univariate analysis

DM = diabetes mellitus; IHD = ischaemic heart disease; eGFR = estimated glomerular filtration rate; RRT = Renal replacement therapy; CCI = Charlson comorbidity index; ESKD = End-stage kidney disease; AKI = Acute kidney injury
